# Protist enteroparasites in wild boar (*Sus scrofa ferus*) and black Iberian pig (*Sus scrofa domesticus*) in southern Spain: a protective effect on hepatitis E acquisition?

**DOI:** 10.1186/s13071-020-04152-9

**Published:** 2020-06-03

**Authors:** Antonio Rivero-Juarez, Alejandro Dashti, Pedro López-López, Aly Salimo Muadica, Maria de los Angeles Risalde, Pamela C. Köster, Isabel Machuca, Begoña Bailo, Marta Hernández de Mingo, Elena Dacal, Ignacio García-Bocanegra, José M. Saugar, Rafael Calero-Bernal, David González-Barrio, Antonio Rivero, Verónica Briz, David Carmena

**Affiliations:** 1 Infectious Diseases Unit, Maimonides Institute for Biomedical Research (IMIBIC), University Hospital Reina Sofía, Córdoba, University of Córdoba, Córdoba, Spain; 2 Parasitology Reference and Research Laboratory, Spanish National Centre for Microbiology, Majadahonda, Madrid Spain; 3 Department of Anatomy and Compared Pathological Anatomy, University of Córdoba, Agrifood Excellence International Campus (ceiA3), Córdoba, Spain; 4 Department of Animal Health, Faculty of Veterinary, University of Córdoba-Agrifood Excellence International Campus (ceiA3), Córdoba, Spain; 5 SALUVET, Department of Animal Health, Faculty of Veterinary, Complutense University of Madrid, Madrid, Spain; 6 Viral Hepatitis Reference and Research Laboratory, Spanish National Centre for Microbiology, Majadahonda, Madrid Spain

**Keywords:** Hepatitis E virus, Enteric parasites, *Cryptosporidium*, *Giardia*, *Blastocystis*, *Strongyloides*, Transmission, Pigs, Wild boars, Co-infection, Spain

## Abstract

**Background:**

Several studies have independently evaluated the occurrence of hepatitis E virus (HEV) and enteroparasites in swine, but no surveys have been conducted to jointly assess the prevalence and genetic diversity of enteroparasites in pigs and wild boars, their sympatric transmission between hosts, and their potential interaction with HEV.

**Methods:**

We prospectively collected serum and faecal samples from black Iberian domestic pigs and wild boars from southern Spain between 2015‒2016. We evaluated for HEV in serum and faeces, and for the presence of enteroparasites (*Giardia duodenalis*, *Cryptosporidium* spp., *Blastocystis* sp., *Neobalantidium coli* and *Strongyloides* spp.) in the same faecal samples. The prevalence of each intestinal parasite species was calculated.

**Results:**

A total of 328 animals (56.7% black Iberian pigs and 43.3% wild boars) were included in the study. The overall global prevalence of HEV in serum was 16.8%. The overall global prevalence of each enteroparasite species was 19.5% for *G. duodenalis*, 8.2% for *Cryptosporidium* spp., 41.8% for *Blastocystis* sp., 31.4% for *N. coli*, and 8.8% for *Strongyloides* spp. HEV-infected animals showed a significantly lower prevalence of *G. duodenalis* (3.2 *vs* 20%; *P* = 0.002) and *Blastocystis* sp. (38.7 *vs* 80%; *P* < 0.001) than those uninfected by HEV. Animals carrying *G. duodenalis* and *Blastocystis* sp. infections showed a significantly lower rate of HEV infection than those not harbouring these enteroparasites (*P* < 0.001).

**Conclusions:**

Our study found a high prevalence of enteroparasites in black Iberian pigs and wild boars in southern Spain, suggesting a sympatric co-transmission of some of the species investigated. It is suggested that extracellular *G. duodenalis* and *Blastocystis* sp. might have a protective effect on HEV acquisition in swine.
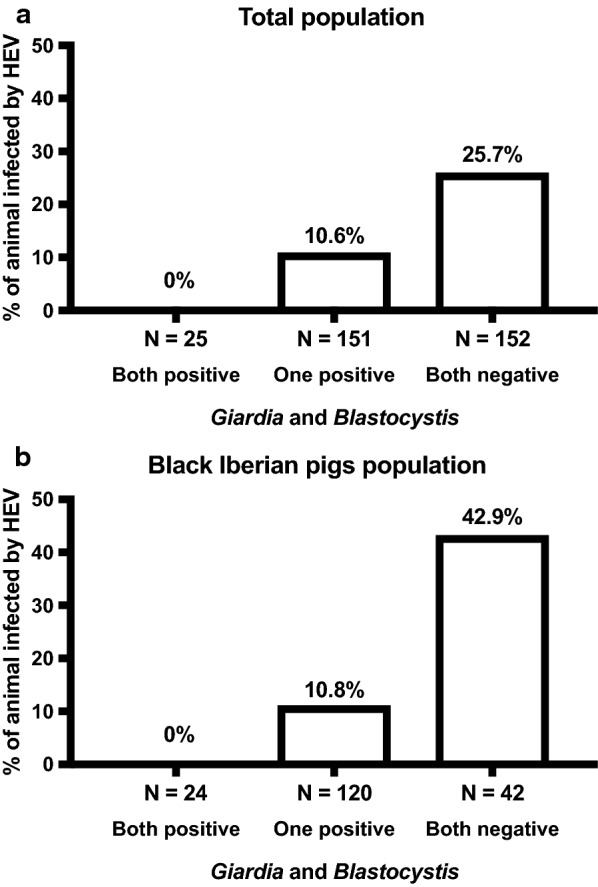

## Background

The intestinal protozoans *Giardia duodenalis* and *Cryptosporidium* spp. are major contributors to the burden of diarrhoeal disease in humans and livestock species globally [1, 2]. Both parasites are transmitted *via* the faecal-oral route either indirectly through ingestion of contaminated water or food or directly by contact with infected persons or animals. Consequently, waterborne and foodborne outbreaks of cryptosporidiosis and giardiosis in humans are frequently reported [3, 4]. The stramenopile *Blastocystis* sp. is the protist most frequently found colonizing/infecting the intestinal tract of humans [5]. Because asymptomatic colonization is very common and pathogenicity has not been conclusively demonstrated *in vivo*, the clinical significance of *Blastocystis* sp. remains controversial [6]. Little is known on the epidemiology of *Blastocystis* sp. in non-human animal species. The ciliate *Neobalantidium coli* is a ubiquitous parasite commonly found in domestic and wild swine, with most cases being asymptomatic [7]. The parasite is transmitted *via* the faecal-oral route primarily through contaminated water with cysts. Humans in close contact with pigs or their excrements may accidentally become infected. Finally, the presence of soil-transmitted helminths belonging to the genus *Strongyloides* seem to be a common finding in free-range pigs raised in low-income countries [8], but little or no information at all is available from swine in high-income countries including Spain.

Hepatitis E virus (HEV) is the leading cause of acute hepatitis worldwide [9]. For this reason, and together with the widespread and relatively easy transmission of the virus, HEV infection is regarded as an emerging major public health problem. HEV is an RNA virus of the family *Hepeviridae* with marked genetic heterogeneity. Five distinct HEV genotypes have been demonstrated to cause human infections; genotypes HEV-1 and HEV-2 infect humans alone, genotypes HEV-3 and HEV-4 primarily infect pigs, wild boars and deer, and genotype HEV-7 primarily infects camelids [10]. This enterically acquired virus is associated with large disease outbreaks associated with the consumption of contaminated water mostly in poor-resource settings characterized by little or no access to safe drinking water, and inadequate sanitation and hygiene [11]. Sporadic cases linked to pork consumption have also been reported in high-income countries [10, 12]. Other sources of infection may include contact with infected organs or carcasses at the time of slaughter or during hunting and evisceration of game, or contact with manure from infected swine.

The main hosts are humans (with a seroprevalence rate ranging between 5‒50%) and pigs (with a seroprevalence rate ranging between 10‒80%) [13, 14]. In most humans and pigs HEV infections present as subclinical and self-limiting hepatitis [12]. Indeed, a worst prognosis might be observed in some populations including pregnant, liver cirrhotic, and immunosuppressed patients [15–17]. Because swine are the main host of HEV, the consumption of raw or under-cooked pork constitutes one of the main routes of transmission [10].

Despite the publication of several studies independently evaluating the prevalence of HEV and enteroparasites in swine, no surveys have been conducted to date to jointly assess the prevalence and genetic diversity of common intestinal parasite (including protozoans, stramenopiles, ciliates and helminths) species in pigs and wild boars, their sympatric transmission between host species, and their potential interaction with HEV.

## Methods

### Study area, sampling and data collection

We prospectively collected serum and feces samples from black Iberian domestic pigs and wild boar from Córdoba (southern Spain) between 2015‒2016. Black Iberians pigs were bred in extensive farm production system, sharing habitat with the wild boar population included in the study. Sampling was conducted to determine prevalence and risk factors for HEV infection. Additional information regarding the methods and procedures involved can be found elsewhere [18, 19]. For the purpose of the present study, we randomly selected black Iberian pigs and wild boars, stratifying the former by age and the latter by age and sex.

Prospectively, we evaluated samples for HEV in serum and faeces [18, 19], and, for the purpose of this study, we retrospectively assessed the presence of enteroparasites in DNA samples. An aliquot (~ 1 g) of faeces was stored at − 80 °C within 24 h of collection until nucleic acid extraction. Extraction and purification of total nucleic acid was carried out from 200 μl of faecal supernatants using the QIAamp Cador Pathogen Mini Kit (Qiagen, Hilden, Germany). Serum samples were obtained from 5 ml of whole blood after centrifugation at 3000×*g* for 10 min. Viral RNA was extracted using the QIAmp Minielute Virus Spin Kit (Qiagen). All nucleic acid extraction procedures were performed using automated equipment (QIAcube, Qiagen). DNA and RNA samples were frozen at − 80 °C until downstream analysis.

#### Molecular detection and characterization of *Giardia duodenalis*

Initial detection of *G. duodenalis* DNA was achieved using a real-time PCR (qPCR) method targeting a 62-bp region of the gene codifying the small subunit ribosomal RNA (*SSU* rRNA) of the parasite [20]. Amplification reactions (25 μl) consisted of 3 μl of template DNA, 0.5 µM of each primer Gd-80F and Gd-127R, 0.4 µM of probe (Additional file 1: Table S1), and 12.5 μl TaqMan® Gene Expression Master Mix (Applied Biosystems, CA, USA). Detection of parasitic DNA was performed on a Corbett Rotor GeneTM 6000 real-time PCR system (Qiagen) using an amplification protocol consisting on an initial hold step of 2 min at 55 °C and 15 min at 95 °C, followed by 45 cycles of 15 s at 95 °C and 1 min at 60 °C. Water (no template) and genomic DNA (positive) controls were included in each PCR run.

*Giardia duodenalis* isolates that tested positive by qPCR were subsequently assessed by sequence-based multi-locus genotyping of the genes encoding for the glutamate dehydrogenase (*gdh*) [21], β-giardin (*bg*) [22], and triose phosphate (*tpi*) [23] proteins of the parasite. Amplifications were conducted by semi-nested and nested PCR protocols using specific primer pairs (Additional file 1: Table S1).

### Molecular detection and characterization of *Cryptosporidium* spp.

The presence of *Cryptosporidium* spp. was assessed using a nested-PCR protocol to amplify a 587 bp fragment of the *SSU* rRNA gene of the parasite [24]. Amplification reactions (50 μl) included 3 μl of DNA sample and 0.3 μM of the primer pairs CR-P1/CR-P2 in the primary reaction and CR-P3/CPB-DIAGR in the secondary reaction (Additional file 1: Table S1). Both PCR reactions were carried out as follows: one step at 94 °C for 3 min, followed by 35 cycles of 94 °C for 40 s, 50 °C for 40 s and 72 °C for 1 min, concluding with a final extension step at 72 °C for 10 min.

### Molecular detection and characterization of *Blastocystis* sp.

Identification of *Blastocystis* sp. was achieved by a direct PCR protocol targeting the *SSU* rRNA gene of the parasite [25]. The assay uses the pan-*Blastocystis*, barcode primer pair RD5/BhRDr to amplify a PCR product of ~600 bp. Amplification reactions (25 μl) included 5 μl of template DNA and 0.5 μM of each primer (Additional file 1: Table S1). Amplification conditions consisted of one step at 95 °C for 3 min, followed by 30 cycles of 1 min each at 94 °C, 59 °C and 72 °C, and a final extension step at 72 °C for 2 min.

### Molecular detection of *Neobalantidium coli*

Detection of *N. coli* was attempted by a direct PCR assay to amplify the complete ITS1-*5.8S*-ITS2 rRNA region and the last 117 bp (3′-end) of the *SSU*-rRNA sequence of this ciliate using the primer set B5D/B5RC [26]. PCR reactions (25 μl) consisted of 2 μl of template DNA and 0.4 μM of each primer (Additional file 1: Table S1). PCR conditions were as follows: 94 °C for 10 min, 30 cycles of 94 °C for 1 min, 60 °C for 1 min, 72 °C for 1 min, and a final extension step at 72 °C for 5 min.

All the direct, semi-nested, and nested PCR protocols described above were conducted on a 2720 Thermal Cycler (Applied Biosystems). Reaction mixes always included 2.5 units of MyTAQ^TM^ DNA polymerase (Bioline GmbH, Luckenwalde, Germany), and 5× MyTAQ^TM^ reaction buffer containing 5 mM dNTPs and 15 mM MgCl_2_. Laboratory-confirmed positive and negative DNA samples for each parasite species investigated were routinely used as controls and included in each round of PCR. PCR amplicons were visualized on 2% D5 agarose gels (Conda, Madrid, Spain) stained with Pronasafe nucleic acid staining solution (Conda). Positive PCR products were directly sequenced in both directions using appropriate internal primer sets (Additional file 1: Table S1). DNA sequencing was conducted by capillary electrophoresis using BigDye® Terminator chemistry (Applied Biosystems) on an on ABI PRISM 3130 Genetic Analyzer.

### Molecular detection of *Strongyloides* spp

Identification of *Strongyloides* spp. was carried out by a qualitative qPCR method using genus-specific primers targeting a 101 bp fragment of the *SSU* rRNA gene of the parasite [27] using SybrGreen reagents (Invitrogen, San Diego CA, USA) as described elsewhere [28]. qPCR reactions (25 µl) contained 10 µl of template DNA, 0.2 µM of each primer Stro18S-1530F/Stro18S-1630R (Additional file 1: Table S1), 1× Quantimix EasyMaster mix (Biotools B&M Laboratories, Madrid, Spain), and 0.5 µl of 50× SybrGreen (Invitrogen). All DNA specimens were assayed in duplicate. Positive, negative, and no template controls were included in each run. The amplification program consisted of 15 min at 95 °C, followed by 50 cycles of 10 s at 95 °C, 10 s at 60 °C and 30 s at 72 °C. DNA amplification and data analyses were as described above for the detection of *G. duodenalis*.

The sequences obtained in this study have been deposited in GenBank under accession numbers MT108431–MT108433 (*G. duodenalis*), MT112069–MT112074 (*N. coli*), MT114474–MT114479 (*Cryptosporidium* spp.) and MT114480–MT114489 (*Blastocystis* sp.).

### Statistical analysis

The prevalence of each intestinal parasite species in the surveyed black Iberian pig and wild boar populations was calculated from the proportion of positive samples with respect to the total number of samples analysed with 95% confidence interval (95% CI). The prevalence was also calculated by animal type (sows and fattening pigs, in the case of black Iberian pigs), sex and age group. These categorical variables were expressed as percentages and the frequencies were compared using the Chi-square or Fisher’s tests. The statistical significance was established at a *P*-value < 0.05. A logistic regression model for HEV infection was performed in the whole population and sorting population in black Iberian pigs and wild boars. Analyses were carried out using SPSS statistical software package version 18.0 (IBM Corporation, Somers, NY, USA).

## Results

### Study population

A total of 328 animals were included in the study. Of these, 56.7% (186/328) were black Iberian pigs, and 43.3% (142/328) wild boars. Among pigs, 52.7% (98/186) were sows and 47.3% (88/186) fattening individuals. Respective to wild boars, 42.8% (48/112) were males and 57.2% (64/112) females. However, sex was unknown for 30 animals. According to age, 47.2% (67/142) were juveniles and 52.8% (75/142) adults.

### Prevalence of HEV and enteroparasites

The overall prevalence of HEV in serum and feces was 16.8% (55/328) and 9.1% (30/328), respectively. No statistically significant differences were observed between wild boars and black Iberian pigs in prevalence of HEV in serum (16.9 *vs* 16.7%; *P* = 0.99) or feces (7.7 *vs* 10.2%; *P* = 0.563). In total, 73 (22.3%) of the animals were positive for HEV in serum and/or feces [22.6% (42/186) black Iberians pigs and 21.8% (31/142) wild boars (*P* = 0.894)]. As previously described, differences in HEV prevalence were not found in relation to age and sex in both black Iberian pigs and wild boars host [18, 19].

In Table 1, the PCR-based prevalence rates for *G. duodenalis*, *Cryptosporidium* spp., *Blastocystis* sp., *N. coli* and *Strongyloides* spp. are shown. Pigs had a higher prevalence of *Blastocystis* sp. (73.1 *vs* 0.7%; *P* < 0.001), *N. coli* (52.7 *vs* 11.7%; *P* < 0.001), and *Strongyloides* spp. (11.8 *vs* 4.9%; *P* = 0.032) than wild boars. Among black Iberian pigs, the prevalence of *G. duodenalis* was higher in sows than in fattening pigs (29.5 *vs* 6.1%; *P* < 0.001). In contrast, the prevalence of *Blastocystis* sp. was higher in fattening pigs than in sows (81.6 *vs* 63.6%; *P* = 0.008). Regarding wild boars, males were significantly more infected by *N. coli* than females (23.3 *vs* 7.8%; *P* = 0.044) and adults than non-adults (18.3 *vs* 4.5%; *P* = 0.016). In this host population, the prevalence of *G. duodenalis* was significantly higher in non-adults than in adults (34.3 *vs* 12.0%; *P* = 0.002).

**Table 1:** Prevalence of enteroparasite species in the studied swine (black Iberian pig and wild boar) populations

Species/subtype	Total population (*n* = 328)	Black Iberian pigs (*n* = 186)	Wild boars (*n* = 142)	*P-*value
	*n* (%)	*n* (%)	*n* (%)	
*Giardia duodenalis*	64 (19.5)	32 (17.2)	32 (22.5)	0.261
*Cryptosporidium* spp.	27 (8.2)	18 (9.7)	9 (6.3)	0.351
*C. scrofarum*	26 (96.2)	18 (100)	8 (88.8)	0.216
*C. suis*	1 (3.8)	0 (0)	1 (11.2)	
*Blastocystis* sp.	137 (41.8)	136 (73.1)	1 (0.7)	**< 0.001**
ST1	7 (5.1)	7 (5.1)	0 (0)	0.99
ST3	20 (14.7)	20 (14.7)	0 (0)	
ST5	110 (80.2)	109 (80.1)	1 (100)	
*Neobalantidium coli*^a^	103 (31.4)	87 (52.7)	16 (11.7)	**< 0.001**
*Strongyloides* spp.	29 (8.8)	22 (11.8)	7 (4.9)	**0.032**

^a^Available in 302 individuals

*Note*: Bold values denote statistical significance at the *P* < 0.05 level

### Molecular characterization of detected enteroparasites

Swine *G. duodenalis*-positive samples by qPCR generated quantification cycle (Cq) values ranging from 26.0 to 41.2 (median: 35.2). Of these, 9.4% (6/64) produced Cq values between 25–30, 34.4% (22/64) between > 30–35, and 56.2% (36/64) > 35. Out of the 32 *G. duodenalis*-positive samples from black Iberian pigs only 3 were successfully genotyped, revealing the presence of assemblages A (*n* = 1) and E (*n* = 2) at the *gdh* and/or *bg* (but not the *tpi*) loci (Table 2). These three samples had qPCR Cq values of 26.0, 28.3 and 30.8, respectively. The sample assigned to assemblage A belonged to the sub-assemblage AI (Additional file 2: Table S2). None of the 32 *G. duodenalis*-positive samples from wild boars could be genotyped.

**Table 2:** Diversity and frequency of parasite species/genotypes identified in the black Iberian pig and wild boar population investigated in the present study

Host species	Parasite species	Genotype	*n*
Black Iberian pig	*Giardia duodenalis*	A	1
		E	2
	*Cryptosporidium scrofarum*	‒	18
	*Blastocystis* sp.	ST1	7
		ST3	20
		ST5	109
Wild boar	*Cryptosporidium scrofarum*	‒	8
	*Cryptosporidium suis*	‒	1
	*Blastocystis* sp.	ST5	1

*Abbreviation*: n, number of isolates

*Cryptosporidium scrofarum* was the only *Cryptosporidium* species detected in black Iberian pigs (Table 2). Out of the 18 sequences analysed, 17 were identical to the GenBank reference sequence KF597530, whereas one showed a single nucleotide polymorphism (SNP) at position 440 (C/T) (Additional file 3: Table S3). Two *Cryptosporidium* species were found circulating in wild boars: *C. scrofarum* (*n* = 8) and *C. suis* (*n* = 1) (Table 2). Six of the *C. scrofarum* sequences showed 100% homology with GenBank sequence KF597530, whereas two had a single SNP at positions 403 (A/W) and 441 (A/G), respectively (Additional file 3: Table S3). The *C. suis* sequence differed from murine *C. occultus* (GenBank reference sequence MG699179) by a deletion in 486-489_DelATTA (Additional file 3: Table S3).

Three *Blastocystis* subtypes (ST) were identified in the 136 black Iberian pigs that carried the parasite, ST1 (5.1%), ST3 (14.7%) and ST5 (80.2%) (Table 2). Further sequence analyses revealed the presence of allele 4 within ST1, alleles 34 and 52 within ST3, and alleles 16, 17, 115, 119, 16+17, and 115+119 within ST5. Allele 119 was the most prevalent (69.3%), followed by allele 34 (4.4%), and allele 4 (3.6%) (Additional file 4: Table S4). *Blastocystis* sp. was only found in a wild boar that carried ST5 allele 115. This very same allele was also detected in a single black Iberian pig.

### Interaction between HEV and enteroparasites

We investigated the interaction between HEV and enteroparasite carriage/infections (Table 3). Animals infected by HEV showed a significantly lower prevalence of *G. duodenalis* (3.2 *vs* 20%; *P* = 0.002) and *Blastocystis* sp. (39 *vs* 80%; *P* < 0.001) than those animals uninfected by HEV. Interestingly, this association was observed with detectable HEV in serum but not in faeces, suggesting a role on active HEV infection. By multivariate analysis, we identified carriage/infection with *G. duodenalis* and *Blastocystis* sp. as a protective factor associated with detectable HEV RNA in serum both in the global swine (black Iberian pigs and wild boars) population and in the black Iberian pig population alone (Table 4). Of note, no significant associations were demonstrated between HEV and the intracellular parasite *Cryptosporidium* spp. In wild boars, the only factor associated with HEV infection was male sex.

**Table 3:** Prevalence of detectable HEV RNA in faeces, serum, and overall, according to presence of the enteroparasite species analysed

Positive for	HEV+	HEV−	*P-*value	HEVs+	HEVs−	*P-*value	HEVf+	HEVf−	*P-*value
	*n* (%)	*n* (%)		*n* (%)	*n* (%)		*n* (%)	*n* (%)	
Global swine population									
*Giardia duodenalis*	8 (11.0)	56 (22.0)	0.044	4 (7.3)	60 (22)	**0.014**	4 (13.3)	60 (20.1)	0.473
*Cryptosporidium* spp.	7 (9.6)	20 (7.8)	0.632	5 (9.1)	22 (8.1)	0.789	3 (10.0)	24 (8.1)	0.725
*Blastocystis* sp.	22 (30.1)	115 (45.1)	**0.023**	12 (21.8)	125 (45.8)	**0.001**	15 (50.0)	122 (40.9)	0.340
*Neobalantidium* spp.^a^	23 (32.9)	80 (34.5)	0.886	17 (32.1)	86 (34.5)	0.873	9 (31.0)	94 (34.4)	0.838
*Strongyloides* spp.	5 (6.8)	24 (9.4)	0.642	3 (5.5)	26 (9.5)	0.440	3 (10.0)	26 (8.7)	0.738
Black Iberian pigs									
*Giardia duodenalis*	3 (7.1)	29 (20.1)	0.062	1 (3.2)	31 (20.0)	**0.020**	2 (10.5)	30 (18.0)	0.537
*Cryptosporidium* spp.	3 (7.1)	15 (10.4)	0.768	3 (9.7)	15 (9.7)	0.990	1 (5.3)	17 (10.2)	0.700
*Blastocystis* sp.	22 (52.4)	114 (79.2)	0.001	12 (38.7)	124 (80.0)	**< 0.001**	15 (78.9)	121 (72.5)	0.785
*Neobalantidium* spp.^b^	18 (43.9)	69 (55.6)	0.210	13 (41.9)	74 (55.2)	0.232	8 (44.4)	79 (53.7)	0.467
*Strongyloides* spp.	4 (9.5)	18 (12.5)	0.788	3 (9.7)	19 (12.3)	0.990	2 (10.5)	20 (12.0)	0.990
Wild boars									
*Giardia duodenalis*	5 (16.1)	27 (24.3)	0.467	3 (12.5)	29 (24.6)	0.285	2 (18.2)	30 (22.9)	0.990
*Cryptosporidium* spp.	4 (12.9)	5 (4.5)	0.105	2 (8.3)	7 (5.9)	0.648	2 (18.2)	7 (5.3)	0.146
*Blastocystis* sp.	0 (0)	1 (0.9)	0.990	0 (0)	1 (0.8)	0.990	0 (0)	1 (0.8)	0.990
*Neobalantidium* spp.^c^	5 (17.2)	11 (10.2)	0.330	4 (18.2)	12 (10.4)	0.290	1 (9.1)	15 (11.9)	0.990
*Strongyloides* spp.	1 (3.2)	6 (5.4)	0.990	0 (0)	7 (5.9)	0.602	1 (9.1)	6 (4.6)	0.439

^a^Available in 302 swines

^b^Available in 165 black Iberian pigs

^c^Available in 137 wild boars

*Note*: Bold values denote statistical significance at the *P* < 0.05 level

*Abbreviations*: HEV, hepatitis E virus; HEVs+, positive serum samples for hepatitis E virus; HEVf+, positive faecal samples for hepatitis E virus

**Table 4:** Multivariate analysis on HEV infection in the global, black Iberian pig, and wild boar populations

Variable	Condition	OR	95% CI	*P-*value
Global swine population				
*Giardia duodenalis*	Negative	0.224	0.06‒0.754	**0.016**
*Cryptosporidium* spp.	Negative	1.062	0.334‒3.377	0.918
*Blastocystis* sp.	Negative	0.298	0.133‒0.665	**0.003**
*Neobalantidium* spp.	Negative	1.474	0.703‒3.089	0.304
*Strongyloides* spp.	Negative	0.862	0.238‒3.126	0.822
Black Iberian pigs				
*Giardia duodenalis*	Negative	0.125	0.015‒1.036	0.054
*Cryptosporidium* spp.	Negative	2.182	0.489‒9.736	0.307
*Blastocystis* sp.	Negative	0.163	0.06‒0.421	**< 0.001**
*Neobalantidium* spp.	Negative	0.874	0.336‒2.273	0.783
*Strongyloides* spp.	Negative	1.073	0.263‒4.383	0.921
Type of pig	Sow	2.148	0.851‒5.421	0.105
Wild boars				
*Giardia duodenalis*	Negative	0.778	0.081‒7.428	0.328
*Cryptosporidium* spp.	Negative	1.011	0.1‒10.254	0.313
*Blastocystis* sp.	Negative	–	–	–
*Neobalantidium* spp.	Negative	1.125	0.295‒4.29	0.476
*Strongyloides* spp.	Negative	–	–	–
Sex	Male	2.940	1.028‒8.408	**0.034**
Age	Juvenile	1.042	0.335‒3.235	0.356

*Note*: Bold values denote statistical significance at the *P* < 0.05 level

*Abbreviations*: OR, odds ratio; CI, confidence interval

**Fig. 1:**
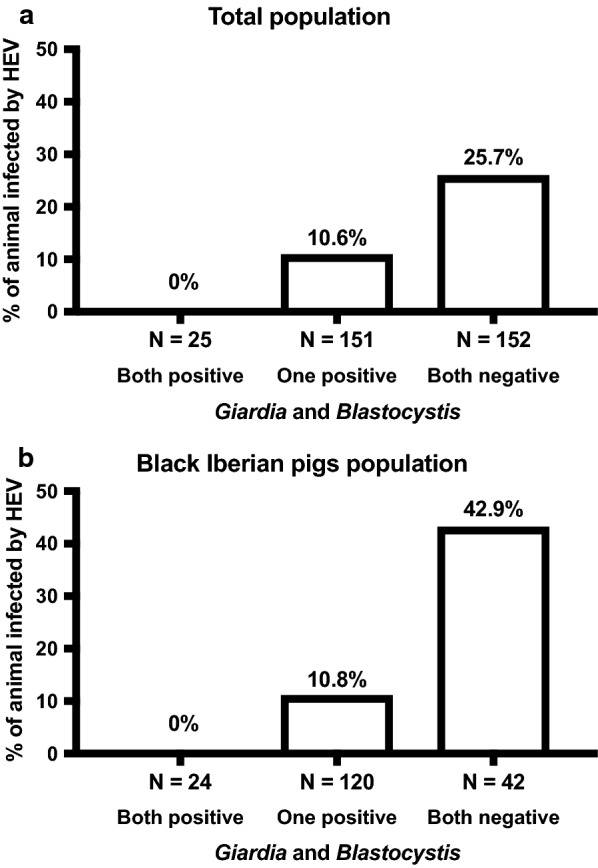
Prevalence of detectable hepatitis E virus RNA in serum in the overall population (**a**) and black Iberian pigs (**b**) according to *Giardia duodenalis* and *Blastocystis* sp. status

Animals were grouped according to *G. duodenalis* and *Blastocystis* sp. status as: (i) positive for both enteroparasites; (ii) positive for at least one enteroparasite; and (iii) negative for both enteroparasites. Overall, 46.3% (152/328) of the tested animals were negative for both *G. duodenalis* and *Blastocystis* sp., 46.0% (151/328) animals tested positive for one of them, and 7.6% (25/328) were positive for both eukaryotes. The prevalence of detectable HEV RNA in serum in these three groups is shown for the total swine (Fig. 1a) and in the black Iberian pig (Fig. 1b) populations. Animals with detectable *G. duodenalis* and *Blastocystis* sp. showed a significantly lower rate of HEV infection than those not bearing these enteroparasites (*P* < 0.001).

## Discussion

Regarding the occurrence of intestinal parasites, the protozoan *G. duodenalis* and *Cryptosporidium* spp. were detected at similar rates in the investigated swine populations (17 *vs* 23% and 6 *vs* 10%, respectively). Based on the qPCR Cq values obtained, *G. duodenalis* seems to be present mostly as a light infection. In Spain, *Cryptosporidium* infections have been reported by microscopy examination at rates of 23% in farmed pigs in north-eastern Spain [29], and of 18% in wild boar populations in the Galicia autonomous region (north-western Spain) [30, 31]. *Giardia duodenalis* infections/cysts have been identified in wild boars from the latter Spanish region at a rate of 8% [30], and in slurry from intensive pig farms [32]. In our study *Blastocystis* sp. was very common in black Iberian pigs but almost absent in wild boars (73 *vs* 0.7%). Additionally, the *Blastocystis* sp. prevalence observed in our black Iberian pig population was considerably higher than those (7‒47%) previously described in intensively reared pigs in other Spanish regions [33, 34].

Following the same trend observed for *Blastocystis* sp. (although to a lesser extent) infection rates of *N. coli* and *Strongyloides* spp. were substantially lower in wild boars compared with those observed in black Iberian pigs. *Neobalantidium coli* is widely recognized as a very common opportunistic parasite of domestic pigs, where infections are mostly subclinical (balantidial dysentery is rare), and is therefore considered of limited veterinary relevance in high-income countries [7]. This is, to the best of our knowledge, the first PCR-based report of *N. coli* in wild boars in Spain. The finding of *Strongyloide*s spp. in both swine populations was also interesting. Porcine strongyloidosis by *S. suis* (syn. *S. ransomi*) is known to infect domestic pigs and wild boars globally, usually at low levels [35].

Regarding molecular data, this is, to the best of our knowledge, the first report of *G. duodenalis* assemblages A and E in Spanish black Iberian pigs. Two *Cryptosporidium* species were identified. Black Iberian pigs were exclusively infected by *C. scrofarum*, whereas wild boars harboured both *C. scrofarum* and *C. suis*. Both species have been previously identified in asymptomatic farmed pigs in north-eastern Spain [29], and *C. suis* in wild boars in north-western Spain [31]. This study also provides the first molecular data on the occurrence and genetic diversity of *Blastocystis* sp. in black Iberian pigs. As expected, ST5 was identified as the most prevalent genetic variant of the parasite, followed by the zoonotic ST3 and ST1. Both ST1 and ST3 have been identified in asymptomatic, mostly paediatric, populations in Spain [36, 37]. Of interest, the finding of ST5 in the only wild boar demonstrated to carry the parasite may be indicative of transmission between black Iberian pigs and wild boars in this geographical area.

The most important contribution of this study is the striking interaction observed between HEV and intestinal extracellular parasites. Specifically, both *G. duodenalis* and *Blastocystis* sp. seem to modulate the rate of HEV infection (or *vice versa*) in swine in general, and in Black Iberian pigs in particular. Such association was not observed between HEV and the intracellular parasite *Cryptosporidium* spp. At present we cannot demonstrate the directionality of this interaction, but we favour the hypothesis of parasites modulating viral infection based on previous reports in the literature. A similar association was found in a study conducted in Israel, where *G. duodenalis* (but not *Cryptosporidium* spp.) modulated the severity of infection with some enteric pathogens in children under two years of age [38]. In that survey, episodes with rotavirus alone were more severe as compared to episodes when rotavirus was in a co-infection with *G. duodenalis*. The reason for this possible association is completely unknown.

It has been demonstrated that several microorganisms could impair the establishment of the infection by other pathogens. This is the case of the GB virus competing with HIV for CD4+ receptors in the surface of lymphocytes. This rivalry impairs the efficiency of the HIV cycle, delaying the progression of the disease to AIDS [39]. In this sense, our results could fit with this mechanism. We hypothesize a competition between intestinal parasites and HEV as a possible explanation of the modulation of the rate of infection, through a biochemical or physical barrier. In fact, a recent study suggests that *G. duodenalis* can reduce colitis by *Escherichia coli* through the expression and secretion of antimicrobial peptides by the host’s intestinal epithelial cells *via* the release of parasite’s cathepsin B-like cysteine protease that directly inhibits the growth of the bacteria [40]. In addition, *G. duodenalis* infection has been shown to modulate host pro-inflammatory responses to pathogenic bacteria and pro-inflammatory stimuli. For instance, the parasite was able to reduce granulocyte infiltration in an *in vivo* model of bacterial toxin-induced colitis and attenuate inflammation in human intestinal tissue [41]. Secretion of *G. duodenalis* cathepsin B cysteine proteases can also attenuate secretion of the neutrophil chemoattractant interleukin-8 in human small intestinal mucosal tissues activated through administration of *Salmonella enterica* serovar Typhimurium [42]. Finally, *G. duodenalis* has been demonstrated to inhibit epithelial nitric oxide production, a common host defence against pathogen infection, by consuming arginine, the crucial substrate to generate the compound [43]. Studies evaluating this possible protective effect of enteroparasites on HEV infection are warranted. Next-generation sequencing data using the *16S* and *18S* rRNA genes can provide a deeper insight into potential interactions between these pathogens and the host-associated microbial communities.

It should be noted that conclusions drawn in this survey may be biased by some factors. First, although relatively large sample sizes of black Iberian pigs and wild boars were analysed, the limited number of positive cases identified for some enteroparasites (*Cryptosporidium* spp. and *Strongyloides* spp.) may hinder the significance of associated risk factors. Secondly, the *Strongyloides* qPCR method used here, originally used to identify *S. stercoralis* in human faecal samples with genus-specific primers, has not been systematically tested with faecal material from non-human sources. This fact, together with the lack of sequencing data (the 101-bp amplicon is not informative enough to discriminate among different *Strongyloides* species) does not allow as to accurately determine the species of this nematode involved in the infections. Thirdly, we did not quantify the *G. duodenalis* and *Blastocystis* sp. burden in the investigated hosts. It would be very interesting to see how parasite burdens correlate with HEV infection rates. This question should be addressed in future surveys.

## Conclusions

Our study found a high prevalence of enteroparasites in pigs and wild boars from southern Spain. The genetic diversity analyses carried out strongly suggest the sympatric co-transmission of some of the species investigated (*Cryptosporidium scrofarum* and, to a lesser extent, *Blastocystis* sp.). It is hypothesized that extracellular *G. duodenalis* and *Blastocystis* sp. might have a protective effect on HEV acquisition in swine. The extent and directionality of this finding needs to be confirmed and further investigated in well-designed case-control studies.

## Supplementary information

**Additional file 1: Table S1.** Oligonucleotides used for the molecular identification and/or characterization of the intestinal protist and helminth parasites investigated in the present study.

**Additional file 2: Table S2.** Diversity, frequency, and main molecular features of *G. duodenalis* isolates in swine samples. GenBank accession numbers of representative sequences are provided. Novel genotypes are underlined.

**Additional file 3: Table S3.** Diversity, frequency, and main molecular features of *Cryptosporidium* isolates at the *SSU* rRNA loci in swine samples. GenBank accession numbers of representative sequences are provided. Novel genotypes are underlined.

**Additional file 4: Table S4.** Diversity and frequency of *Blastocystis* sp. subtypes and *18S* alleles identified in the present study.

## Data Availability

All relevant data are within the article and its additional files. The sequence data were submitted to the GenBank database under the accession numbers MT108431–MT108433, MT112069–MT112074, and MT114474–MT114489.

## Abbreviations

*bg*: ß-giardin
Cq: quantification cycle
*gdh*: glutamate dehydrogenase
HEV: hepatitis E virus
qPCR: real-time polymerase chain reaction
*SSU* rRNA: small subunit ribosomal RNA
ST: subtype
*tpi*: triose phosphate isomerase
