# Supplementary material for: Protist enteroparasites in wild boar (Sus scrofa ferus) and black Iberian pig (Sus scrofa domesticus) in southern Spain: a protective effect on hepatitis E acquisition?
Source: Parasit Vectors. 2020 Jun 3;13:281. doi: 10.1186/s13071-020-04152-9 (PMC7271453; doi:10.1186/s13071-020-04152-9)
Supplement: Supplementary file 1 — Additional file 1: Table S1. Oligonucleotides used for the molecular identification and/or characterization of the intestinal protist and helminth parasites investigated in the present study. [file 13071_2020_4152_MOESM1_ESM.docx]

**Additional file 1: Table S1** Oligonucleotides used for the molecular identification and/or characterization of the intestinal protist and helminth parasites investigated in the present study

| **Target organism** | **Locus** | **Oligonucleotide** | **Sequence (5'–3')** | **Reference** |
| --- | --- | --- | --- | --- |
| *Giardia duodenalis* | *ssu* rRNA | Probe | FAM–CCCGCGGCGGTCCCTGCTAG–BHQ1 | Verweij et al. (2003) |
|  |  | Gd-80F | GACGGCTCAGGACAACGGTT | Verweij et al. (2003) |
|  |  | Gd-127R | TTGCCAGCGGTGTCCG | Verweij et al. (2003) |
|  | *gdh* | GDHeF | TCAACGTYAAYCGYGGYTTCCGT | Read et al. (2004) |
|  |  | GDHiF | CAGTACACCTCYGCTCTCGG | Read et al. (2004) |
|  |  | GDHiR | GTTRTCCTTGCACATCTCC | Read et al. (2004) |
|  | *bg* | G7_F | AAGCCCGACGACCTCACCCGCAGTGC | Lalle et al. (2005) |
|  |  | G759_R | GAGGCCGCCCTGGATCTTCGAGACGAC | Lalle et al. (2005) |
|  |  | G99_F | GAACGAACGAGATCGAGGTCCG | Lalle et al. (2005) |
|  |  | G609_R | CTCGACGAGCTTCGTGTT | Lalle et al. (2005) |
|  | *tpi* | AL3543 | AAATIATGCCTGCTCGTCG | Sulaiman et al. (2003) |
|  |  | AL3546 | CAAACCTTITCCGCAAACC | Sulaiman et al. (2003) |
|  |  | AL3544 | CCCTTCATCGGIGGTAACTT | Sulaiman et al. (2003) |
|  |  | AL3545 | GTGGCCACCACICCCGTGCC | Sulaiman et al. (2003) |
| *Cryptosporidium* spp. | *ssu* rRNA | CR-P1 | CAGGGAGGTAGTGACAAGAA | Tiangtip & Jongwutiwes (2002) |
|  |  | CR-P2 | TCAGCCTTGCGACCATACTC | Tiangtip & Jongwutiwes (2002) |
|  |  | CR-P3 | ATTGGAGGGCAAGTCTGGTG | Tiangtip & Jongwutiwes (2002) |
|  |  | CPB-DIAGR | TAAGGTGCTGAAGGAGTAAGG | Tiangtip & Jongwutiwes (2002) |
| *Blastocystis* spp. | *ssu* rRNA | BhRDr | GAGCTTTTTAACTGCAACAACG | Scicluna et al. (2006) |
|  |  | RD5 | ATCTGGTTGATCCTGCCAGT | Scicluna et al. (2006) |
| *Neobalantidium coli* | ITS | B5D | GCTCCTACCGATACCGGGT | Ponce-Gordo et al. (2011) |
|  |  | B5RC | GCGGGTCATCTTACTTGATTTC | Ponce-Gordo et al. (2011) |
| *Strongyloides stercoralis* | *ssu* rRNA | Stro18S-1530F | GAATTCCAAGTAAACGTAAGTCATTAGC | Verweij et al. (2009) |
|  |  | Stro18S-1630R | TGCCTCTGGATATTGCTCAGTTC | Verweij et al. (2009) |

*bg*: β-giardin (bg); *gdh*: Glutamate dehydrogenase; ITS: Internal transcribed spacer; *ssu* rRNA: Small subunit ribosomal RNA; *tpi*: Triose phosphate isomerase.

**References**

Lalle M, Pozio E, Capelli G, Bruschi F, Crotti D, Cacciò SM. Genetic heterogeneity at the beta-giardin locus among human and animal isolates of *Giardia duodenalis* and identification of potentially zoonotic subgenotypes. Int J Parasitol*.* 2005;35:207–213. <https://doi.org/10.1016/j.ijpara.2004.10.022>

Ponce-Gordo F, Fonseca-Salamanca F, Martínez-Díaz RA.. Genetic heterogeneity in internal transcribed spacer genes of *Balantidium coli* (Litostomatea, Ciliophora). Protist 2011;162:774–794. <https://doi.org/10.1016/j.protis.2011.06.008>

Read CM, Monis PT, Thompson RC. Discrimination of all genotypes of *Giardia duodenalis* at the glutamate dehydrogenase locus using PCR-RFLP. Infect Genet Evol 2004;4:125–130. <https://doi.org/10.1016/j.meegid.2004.02.001>

Scicluna SM, Tawari B, Clark CG. DNA barcoding of *Blastocystis*. Protist 2006;157:77–85. <https://doi.org/10.1016/j.protis.2005.12.001>

Sulaiman IM, Fayer R, Bern C, Gilman RH, Trout JM, Schantz PM, et al. Triosephosphate isomerase gene characterization and potential zoonotic transmission of *Giardia duodenalis*. Emerg Infect Dis. 2003;9:1444–1452. <https://doi.org/10.3201/eid0911.030084>

Tiangtip R, Jongwutiwes S. Molecular analysis of *Cryptosporidium* species isolated from HIV-infected patients in Thailand. Trop Med Int Health 2002;7:357–364. <https://doi.org/10.1046/j.1365-3156.2002.00855.x>

Verweij JJ, Canales M, Polman K, Ziem J, Brienen EA, Polderman AM, et al. Molecular diagnosis of *Strongyloides stercoralis* in faecal samples using real-time PCR. Trans R Soc Trop Med Hyg. 2009;103:342‒346. <https://doi.org/10.1016/j.trstmh.2008.12.001>

Verweij JJ, Schinkel J, Laeijendecker D, van Rooyen MA, van Lieshout L, Polderman AM. Real-time PCR for the detection of *Giardia lamblia*. Mol Cell Probes 2003;17:223–225. <https://doi.org/10.1016/S0890-8508(03)00057-4>
