# Supplementary material for: Protist enteroparasites in wild boar (Sus scrofa ferus) and black Iberian pig (Sus scrofa domesticus) in southern Spain: a protective effect on hepatitis E acquisition?
Source: Parasit Vectors. 2020 Jun 3;13:281. doi: 10.1186/s13071-020-04152-9 (PMC7271453; doi:10.1186/s13071-020-04152-9)
Supplement: Supplementary file 2 — Additional file 2: Table S2. Diversity, frequency, and main molecular features of G. duodenalis isolates in swine samples. GenBank accession numbers of representative sequences are provided. Novel genotypes are underlined. [file 13071_2020_4152_MOESM2_ESM.docx]

**Additional file 2: Table S2.** Diversity, frequency, and main molecular features of *Giardia duodenalis* isolates in swine samples. GenBank accession numbers of representative sequences were provided. Novel genotypes were shown underlined

| **Host** | **Assemblage** | **Sub-assemblage** | **No. isolates** | **Locus** | **Reference sequence** | **Stretch** | **Single nucleotide polymorphism** | **GenBank accession number** |
| --- | --- | --- | --- | --- | --- | --- | --- | --- |
| Pig | A | AI | 1 | *gdh* | L40509 |  | T430A | MT108431 |
|  | E | ‒ | 2 | *gdh* | U47632 |  | None | MT108432 |
|  |  |  | 2 | *bg* | Y072729 |  | None | MT108433 |

*gdh*: Glutamate dehydrogenase.
