# Supplementary material for: Protist enteroparasites in wild boar (Sus scrofa ferus) and black Iberian pig (Sus scrofa domesticus) in southern Spain: a protective effect on hepatitis E acquisition?
Source: Parasit Vectors. 2020 Jun 3;13:281. doi: 10.1186/s13071-020-04152-9 (PMC7271453; doi:10.1186/s13071-020-04152-9)
Supplement: Supplementary file 3 — Additional file 3: Table S3. Diversity, frequency, and main molecular features of Cryptosporidium isolates at the SSU rRNA loci in swine samples. GenBank accession numbers of representative sequences are provided. Novel genotypes are underlined. [file 13071_2020_4152_MOESM3_ESM.docx]

**Additional file 3: Table S3.** Diversity, frequency, and main molecular features of *Cryptosporidium* isolates at the *ssu* rRNA loci in swine samples. GenBank accession numbers of representative sequences were provided. Novel genotypes were shown underlined

| **Host** | **Species** | **No. isolates** | **Reference sequence** | **Stretch** | **Single nucleotide polymorphism** | **GenBank accession number** |
| --- | --- | --- | --- | --- | --- | --- |
| Pig | *C. scrofarum* | 17 | KF597530 | 293‒741 | None | MT114474 |
|  |  | 1 | KF597530 | 297‒741 | C440T | MT114475 |
| Wild boar | *C. scrofarum* | 6 | KF597530 | 291‒741 | None | MT114476 |
|  |  | 1 | KF597530 | 304‒741 | A403W | MT114477 |
|  |  | 1 | KF597530 | 284‒741 | A441G | MT114478 |
|  | *C. suis*^a^ | 1 | AF115377 | 600‒937 | 689_DelA, 692-695_DelTTTA | MT114479 |

^a^High homology also with *Cryptosporidium occultus* showing 486-489_DelATTA (reference sequence: MG699179).
