# Supplementary material for: Protist enteroparasites in wild boar (Sus scrofa ferus) and black Iberian pig (Sus scrofa domesticus) in southern Spain: a protective effect on hepatitis E acquisition?
Source: Parasit Vectors. 2020 Jun 3;13:281. doi: 10.1186/s13071-020-04152-9 (PMC7271453; doi:10.1186/s13071-020-04152-9)
Supplement: Supplementary file 4 — Additional file 4: Table S4. Diversity and frequency of Blastocystis sp. subtypes and 18S alleles identified in the present study. [file 13071_2020_4152_MOESM4_ESM.docx]

**Additional file 4: Table S4.** Diversity and frequency of *Blastocystis* sp. subtypes and 18S alleles identified in the present study

| **Host** | **Subtype** | **Allele** | **No. isolates** | **Relative frequency (%)^a^** | **Absolute frequency (%)^b^** | **GenBank accession number** |
| --- | --- | --- | --- | --- | --- | --- |
| Pig | ST1 | 4 | 5 | 71.4 | 3.6 | MT114480 |
|  |  | Unknown | 2 | 28.6 | 1.5 | ‒ |
|  | ST3 | 34 | 6 | 30.0 | 4.4 | MT114481 |
|  |  | 52 | 5 | 25.0 | 3.6 | MT114482 |
|  |  | Unknown | 9 | 45.0 | 6.6 | ‒ |
|  | ST5 | 16 | 1 | 0.9 | 0.7 | MT114483 |
|  |  | 17 | 1 | 0.9 | 0.7 | MT114484 |
|  |  | 115 | 1 | 1.8 | 0.7 | MT114485 |
|  |  | 119 | 95 | 86.4 | 69.3 | MT114486 |
|  |  | 16+17 | 1 | 0.9 | 0.7 | MT114487 |
|  |  | 115+119 | 8 | 7.3 | 5.8 | MT114488 |
|  |  | Unknown | 2 | 1.8 | 1.5 | ‒ |
| Wild boar | ST5 | 115 | 1 | 100 | 0.7 | MT114489 |

^a^Over the total number of samples assigned to a given subtype.

^b^Over the total number (*n* = 137) of samples subtyped.
